# Poster Session II - A228 USING SOCIETAL FACTORS TO PREDICT EPIDEMIOLOGIC STAGES OF INFLAMMATORY BOWEL DISEASE

**DOI:** 10.1093/jcag/gwaf042.228

**Published:** 2026-02-13

**Authors:** L Hracs, J Gorospe, J W Windsor, S Coward, S Okabayashi, G G Kaplan

**Affiliations:** University of Calgary, Calgary, AB, Canada; University of Calgary, Calgary, AB, Canada; University of Calgary, Calgary, AB, Canada; University of Calgary, Calgary, AB, Canada; University of Calgary, Calgary, AB, Canada; University of Calgary, Calgary, AB, Canada

## Abstract

**Background:**

Epidemiologic trends of inflammatory bowel disease (IBD) are described in terms of stages: Stage 1, Emergence (low incidence and prevalence); stage 2, Acceleration in Incidence (rapidly rising incidence and low prevalence); and stage 3, Compounding Prevalence (stabilizing incidence and rapidly rising prevalence). Industrialization, urbanization, and westernization have been associated with rising incidence and prevalence of IBD. However, it remains unclear if these societal factors can serve as proxies for epidemiologic trends in countries with limited population-based data.

**Aims:**

To determine epidemiologic stages of IBD in countries without incidence and prevalence data.

**Methods:**

Stage classifications were previously established over 10 decades (1920s–2020s) for 82 countries with population-based IBD incidence and prevalence data. Five societal factors from the most recent complete decade (2010–2019) were examined: Augmented Human Development Index, obesity rate, percent urbanization, Universal Health Coverage Service Index, and Western Diet Index (Table 1). A gradient-boosted decision tree classification model (XGBoost) with 5-fold cross-validation (CV) and an 80/20 train-test split was trained on the societal factors for the 82 countries with known epidemiologic stage classifications spanning 1920–2024. The fitted model was then applied to 87 countries with only societal factor data to predict their epidemiologic stages for 2010–2019. Decade-level stage classifications for each country were determined by identifying the most frequent (mode) stage classifications among year-level predictions.

**Results:**

The XGBoost model demonstrated a strong, consistent performance (mean CV accuracy = 93.7% [95%CI: 92.0, 95.4]) and achieved a high accuracy of 96.0% (95%CI: 94.0, 97.4) on a held-out test dataset. *F*_*1*_ scores indicate a balanced performance across classes (stage 1 = 0.94, stage 2 = 0.97, stage 3 = 0.95) suggesting that the model accurately distinguished between the three epidemiologic stages. Model inference produced epidemiologic stage classifications for 87 countries during 2010–2019 based solely on societal factor data, highlighting epidemiologic trends of IBD across a majority of global regions (Figure 1).

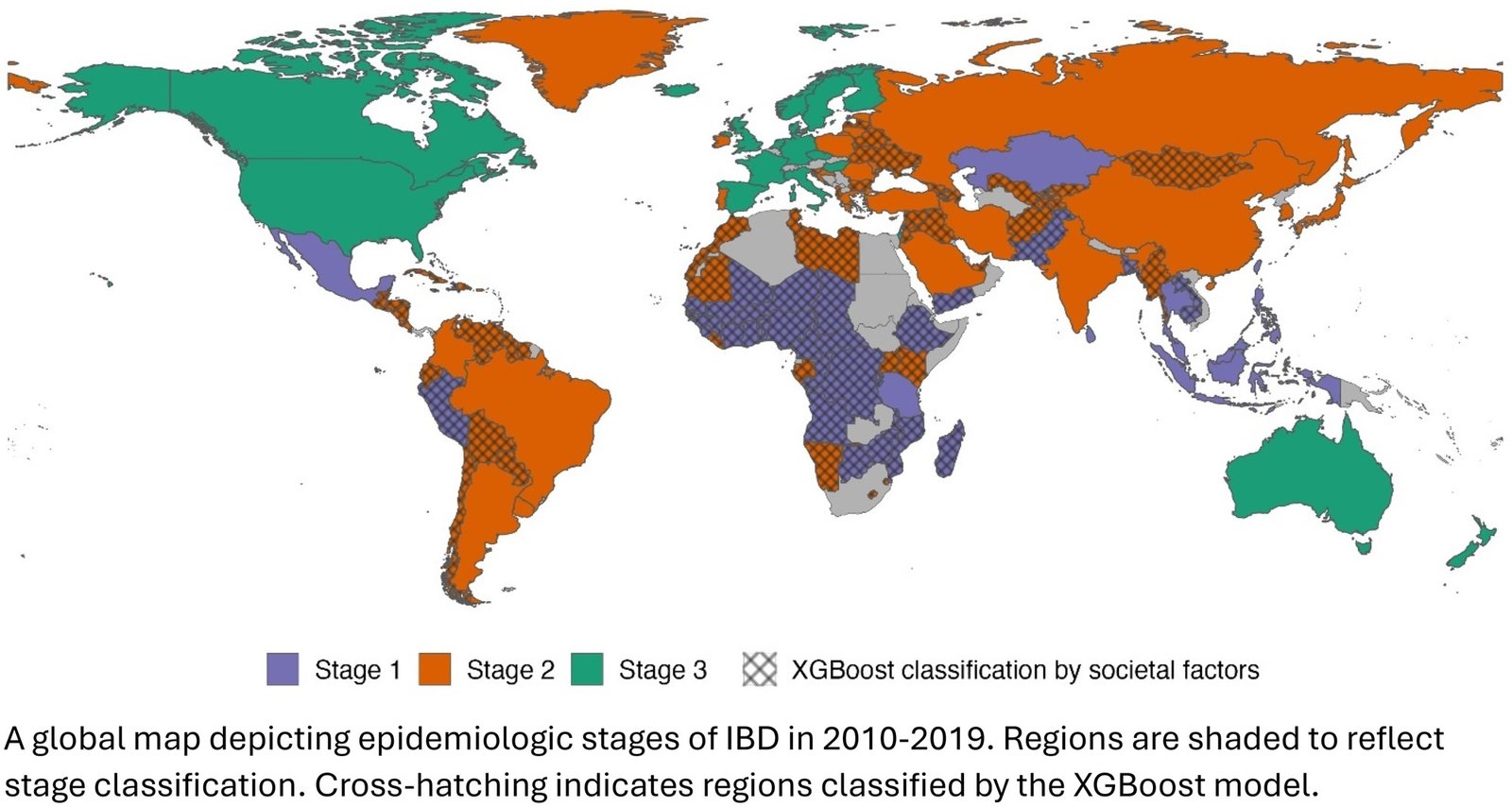

**Conclusions:**

In countries lacking population-based data, societal factors are reliable proxies for epidemiologic trends of IBD. As countries experience economic growth, healthcare advancements, and lifestyle changes, examining societal factors can better prepare global healthcare systems for the evolving burden of IBD.

**Funding Agencies:**

CIHRThe Leona M. and Harry B. Helmsley Charitable Trust; International Organization for the study Inflammatory Bowel Disease (IOIBD)

**A228 Table 1:** Societal factors used in XGBoost classifications.

Augmented Human Development Index	captures the geometric mean of normalized life expectancy, mean years of education, gross domestic product (GDP) per capita, and Varieties of Democracy’s Liberal Democracy Index
obesity rate	a measure of the percentage of an adult population with a BMI >30kg/m2
percent urbanization	a measure of the mid-year percentage of a population living in an urban setting
Universal Health Coverage Service Index	quantifies various aspects of healthcare, including reproductive health (e.g., % of pregnant persons with ≥4 prenatal care visits), prevention of communicable diseases (e.g., % of 1-year-old children with adequate diphtheria, tetanus, and pertussis vaccination), non-communicable diseases (e.g., % cervical cancer screening in females aged 30–49 years), and healthcare access (e.g., number of hospital beds per capita)
Western Diet Index	calculated by dividing the available calories per person per day from animal oils and fats, milk, eggs, plant oils and fats, and sugars by the total available calories per person per day

